# Spreading Processes in Multiplex Metapopulations Containing Different Mobility Networks

**DOI:** 10.1103/PhysRevX.8.031039

**Published:** 2018-08-09

**Authors:** D. Soriano-Paños, L. Lotero, A. Arenas, J. Gómez-Gardeñes

**Affiliations:** ^1^ Departamento de Física de la Materia Condensada, Universidad de Zaragoza, 50009 Zaragoza, Spain and GOTHAM Lab, Instituto de Biocomputación y Física de Sistemas Complejos (BIFI), Universidad de Zaragoza, 50018 Zaragoza, Spain; ^2^ Facultad de Ingeniería Industrial, Universidad Pontificia Bolivariana, Medellín 050031, Colombia; ^3^ Departament d’Enginyeria Informàtica i Matemàtiques, Universitat Rovira i Virgili, 43007 Tarragona, Spain

## Abstract

We propose a theoretical framework for the study of spreading processes in structured metapopulations, with heterogeneous agents, subjected to different recurrent mobility patterns. We propose to represent the heterogeneity in the composition of the metapopulations as layers in a multiplex network, where nodes would correspond to geographical areas and layers account for the mobility patterns of agents of the same class. We analyze classical epidemic models within this framework and obtain an excellent agreement with extensive Monte Carlo simulations. This agreement allows us to derive analytical expressions of the epidemic threshold and to face the challenge of characterizing a real multiplex metapopulation, the city of Medellín in Colombia, where different recurrent mobility patterns are observed depending on the socioeconomic class of the agents. Our framework allows us to unveil the geographical location of those patches that trigger the epidemic state at the critical point. A careful exploration reveals that social mixing between classes and mobility crucially determines these critical patches and, more importantly, it can produce abrupt changes of the critical properties of the epidemic onset.

## Summary

The spread of disease is strongly driven by how humans move and segregate across cities, regions, and countries. Understanding how epidemics arise from the interplay of these behaviors is crucial for implementing efficient containment and prevention policies. In this work, we propose a theoretical framework for the spread of pathogens that incorporates the real patterns of mobility, demographics, and human diversity observed in urban environments.

Our framework represents various populations in a community as layers in a multiplex network, where nodes correspond to geographical areas and layers account for movement patterns. With this framework, we analyze a real urban system, the city of Medellin in Colombia, considering the interplay between six socioeconomic classes displaying disparate demographic segregation and mobility habits. Our framework can identify those neighborhoods and classes that trigger epidemic outbreaks.

Remarkably, we observe that small changes to both mobility and social mixing among these subpopulations can trigger abrupt changes. As a consequence, containment strategies targeting a certain neighborhood can quickly change from efficient to useless. Finally, we provide a physical interpretation of the abrupt changes of those patches driving the unfolding of epidemics based on the effective number of contacts of agents.

Our framework provides a reliable, time-saving platform for analyzing the spread of pathogens among various populations, allowing researchers to identify areas critical to the unfolding of diseases. With further improvements, our formalism could be extended to accommodate more sophisticated commuting patterns and epidemic models.

## I. INTRODUCTION

During the past decades we have witnessed the onset of several major global health threats such as the 2003 spread of severe acute respiratory syndrome (SARS), the H1N1 influenza pandemic in 2009, the western Africa 2014 Ebola outbreaks, and more recently the Zika epidemics in the Americas and Caribbean regions. These outbreaks are increasingly characterized by the small elapsed time between initial infections in a single region to the global epidemic state affecting different cities, regions, countries, and, in some cases, continents. Thus, in recent years a great effort has been devoted to understanding the fast unfolding of emergent diseases and to design both local and global contention strategies. The most common avenue to tackle this problem is to adapt classical epidemic models taking into account the multiscale nature of diseases propagation [1,2].

It is clear that the spread of an emergent infectious disease is the result of human-human interactions in small geographical patches. However, in order to understand the geographical diffusion of diseases, one has to combine these microscopic contagion processes with the long-range disease propagation due to human mobility across different spatial scales. To tackle this problem, epidemic modeling has relied on reaction-diffusion dynamics in metapopulations, a family of models first used in the field of population ecology [3–7]. For the case of epidemic modeling, the usual metapopulation scenario [8–10] is as follows. A population is distributed in a set of patches, with the size (number of individuals) of each patch in principle different. The individuals within each patch are well mixed; i.e., pathogens can be transmitted from an infected host to any of the healthy agents placed in the same patch with the same probability. The second ingredient of metapopulation frameworks concerns the mobility of agents. Each host is allowed to change its current location and occupy another patch, thus fostering the spread of pathogens at the system level. The mobility of agents between different patches is usually represented in terms of a network where nodes are locations while a link between two patches represents the possibility of moving between them.

The nontrivial mobility patterns observed in real populations [11] and recent advances of network epidemiology [12] have motivated a thorough analysis about the impact that the structure of mobility networks has on the onset of global-scale contagions. In the past decade, important steps towards the inclusion of realistic mobility structures have been made [13–16]. These approaches had to compromise between realism and analytical feasibility. On one side, lengthy mechanistic simulations [1,17] provide fair predictions on realistic scenarios while, on the other, theoretical frameworks allowing for analytical results usually rely on strong assumptions limiting their applicability to real-world threats. For instance, it is usual to assume simplified mobility patterns and mean-field approximations for hosts’ and patches’ behavior to be able to predict the onset of an outbreak. In these models, random diffusion of agents between the nodes is often used as a proxy of human mobility while, as in the heterogeneous mean field in contact networks [18], subpopulations with identical connectivity are assumed to be equally affected by the disease.

These mean-field-like approximations for patches having identical properties, while useful for deriving analytical results, add important limitations for their applicability in real-world diseases prediction. As data gathering techniques and epidemic surveillance [19] increase their accuracy, metapopulation models face new challenges [20]. In an effort to overcome the random diffusion of hosts assumption and approach realistic mobility patterns, researchers have recently addressed the recurrent and spatially constrained nature of most human movements [21–27], finding counterintuitive results such as the epidemic detriment caused by mobility [26,27]. However, a theoretical framework of metapopulations of arbitrary structure, incorporating the many aspects of real mobility patterns, remains an open challenge.

One of these aspects to explore is the coexistence, within the same population, of different types of interacting agents and its implications in the spread of pathogens. Previous works along this line have been devoted to incorporate different types of agents according to their age [28] or heterogeneities in terms of infectivities [29,30]. However, those works addressing the interplay between different types of individuals in metapopulations rely on random diffusion of agents [29] or in degree-based assumptions for assigning the occupation of patches and the fluxes between them [30]. Thus, a metapopulation framework incorporating both agents’ heterogeneities and the use of realistic demographic and mobility patterns is still needed, and constitutes the main focus of this work.

To tackle this problem here we draw upon the multiplex formalism, a mathematical representation of networked systems in which different types of interactions between a given set of nodes coexist and interplay. Multiplex networks [31–35] consist of a set of L networks (usually called layers) and a set of N nodes. Each node is represented once in each network layer, allowing it to share different connectivity patterns in each of the L layers. In terms of metapopulation models, for which nodes account for geographical locations, each layer represents the mobility network of each type of agent, while each subpopulation is represented in each layer. This way, the multiplex formalism captures the coexistence, within each subpopulation, of different types of agents with different mobility preferences. These differences can account for age-specific mobility habits (capturing different preferences in the locations visited) or the socioeconomic segregation of residences and work places across urban areas. In these cases, the diverse mobility patterns corresponding to each agent type affect in different ways the onset of epidemics.

In an attempt to increase the realism of epidemiological models without compromising the possibility of a theoretical analysis, here we propose a mathematical framework in which the dynamical variables of each patch forming the metapopulation are treated independently. Our framework can accommodate any mobility multiplex network from real commuting data sets containing different types of individuals and is amenable to any particular distribution of the population across the patches, generalizing previous findings on monolayer networks [26,27]. We analyze the classical susceptible-infected-susceptible (SIS) and the susceptible-infected-recovered (SIR) models, achieving an excellent agreement with intensive Monte Carlo simulations. In addition, we derive an exact expression of the epidemic threshold and show its nontrivial dependence with the different mobility patterns represented in the multiplex.

The multiplex formalism introduced here is suitable to include new realistic factors for modeling spreading processes that former metapopulation models could not account for. As an example of the potential applicability of this formalism, we tackle the spread of diseases over the city of Medellín (Colombia), taking into account that its population is divided into six socioeconomic classes. These classes are represented as a multiplex metapopulation of six layers, each one encoding the demography as well as the mobility patterns of each class. We analyze the spread of diseases over this real configuration and introduce quantifiable measures to shed light on the influence of the social mixing among socioeconomic classes and mobility on the critical properties of the epidemic onset. The interplay between socioeconomical mixing and mobility produces nontrivial effects with strong consequences on the criticality of the epidemics. Specifically, the localization of the epidemics changes abruptly as a consequence of this interplay, having interesting consequences in the design of epidemic containment policies.

## II. METAPOPULATION MODEL

For the sake of clarity, we start by considering a metapopulation framework consisting of one single type of agents. In this case, we have a network composed of N nodes (the patches) and a total population of P agents. Importantly, each agent has associated one of the patches so that all the movements of agents associated to a node, say i, initiate from and return to it. In its turn, a node i of the network is the basement (or home) of a number $n_{i}$ of agents so that $P=\sum_{i=1}^{N}n_{i}$. For the sake of generality, we consider that the network connecting the patches of the population is a weighted and directed graph, encoded in an adjacency matrix whose entries $W_{ij}$ account for the weight of the interaction from node i to node j.

The dynamical model implemented in our metapopulation involves three different stages at each time step t: movement, interaction, and return (MIR). First, each agent decides whether to move with probability p or remain in its associated home node i with probability (1-p). If the agent moves, it goes to any of the nodes connected to i as dictated by the adjacency matrix W. The probability that a patch j is chosen is proportional to the weight of the corresponding entry $W_{ij}$ of the adjacency matrix:

$$R_{ij}=\frac{W_{ij}}{\sum_{j=1}^{N}W_{ij}}.$$ (1)

**FIG. 1.**
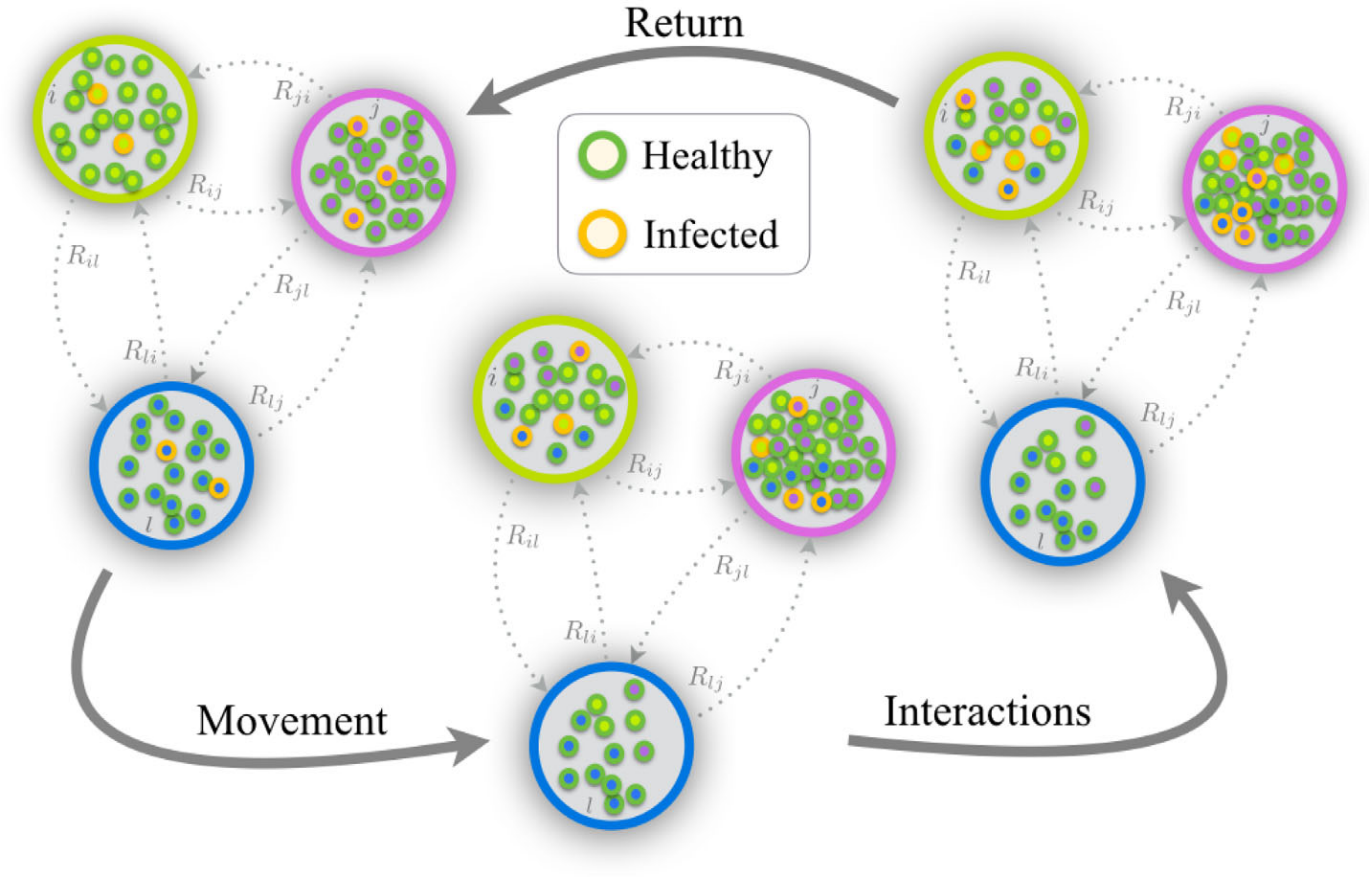
Schematic representation of one time step of the movement-interaction-return (MIR) metapopulation model. The network is composed of N=3 patches. At the *movement* stage some of the local agents decide to move to the other patches according to the probabilities encoded in matrix R. Once agents have moved they interact in a well-mixed way and change their epidemic status (healthy or infected) according to a SIS model. Finally, the agents come back to their home patches and a new time step starts.

Once all the agents have been placed in the nodes, the interaction stage takes place. Each agent updates its dynamical state according to the epidemic model at work (see below) by interacting with the agents that are placed in the same patch at time t. Finally, agents come back to their corresponding residence node and another time step starts. These stages are depicted in Fig. 1 where, for clarity, we have considered that the states of agents are either healthy or infectious, as in the SIS model.

This metapopulation model captures the commuting nature of most of the human displacements within cities (at the level of neighborhoods) or countries (at the level of cities). Interestingly, let us remark that empirical data about real recurrent mobility patterns can be incorporated straightforwardly in the MIR model by considering the number of observed trips between two locations $W_{ij}$ in order to construct the transition rates matrix R. This way, the model has as control parameters the displacement probability p and those controlling the epidemic model under study.

### A. Population-based Markovian dynamics in complex networks

In the following we focus on the two most paradigmatic epidemic models, SIS and SIR. The reaction laws of these models are given by two parameters: (i) the probability λ that a susceptible (healthy) agent catches the diseases after the contact with a single infected individual and (ii) the probability μ that an infected overcomes the disease and turns to be susceptible again (SIS) or becomes immunized (SIR). These reactions can be expressed as

$$S+I\overset{\lambda }{\to }2I,\;I\overset{\mu }{\to }S,$$ (2)

for the SIS model, while for the SIR,

$$S+I\overset{\lambda }{\to }2I,\;I\overset{\mu }{\to }R.$$ (3)

As in any metapopulation model on large complex networks, we face the problem of computationally expensive simulations. A useful avenue to analyze these models, with the by-product of obtaining analytical estimations for the impact of the epidemic, is to formulate coarse-grained models that reduce significantly the complexity of the problem. Typically, heterogeneous mean-field techniques have been applied in a number of works related to epidemic spreading in contact networks and metapopulations. As anticipated above, the main assumption of the heterogeneous mean field is to correlate the relevant parameters of nodes and patches with their number of connections to other nodes, i.e., their degree. This way, two distant patches that are connected to the same number (but not the same set) of locations are considered to have the same static and dynamical properties such as, for instance, the number of habitants and the fraction of infected agents. This assumption, although being strong, has been shown to be valid for small epidemic sizes, thus allowing quite good predictions of epidemic thresholds.

Here we formulate the mathematical equations of the MIR model by following a similar avenue as in Refs. [36–38] for contact networks, thus generalizing the Markovian approach to complex metapopulations. This way, we consider both static and dynamical variables of each individual patch as independent, allowing us to compare directly with the findings of Monte Carlo simulations at the microscopic level and, more importantly, to derive theoretical results for any kind of particular mobility networks.

#### 1. SIS model

For the SIS model, we have a set of N variables $\rho_{i}(t)$ denoting the fraction of infected agents associated to patch i at time t. It is important to stress that, according to the MIR model, an agent whose associated patch is i can be in another node j at time t. The time evolution of $\rho_{i}(t)$ can be written as

$$\rho_{i}(t+1)=(1-\mu )\rho_{i}(t)+[1-\rho_{i}(t)]\Pi_{i}(t),$$ (4)

where the first term denotes the fraction of infected agents associated to i that do not recover at time t+1. The second term instead accounts for the fraction of healthy agents associated to i that pass to infected at time t+1. In this second term, $\Pi_{i}(t)$ is the probability that a healthy agent associated to node i becomes infected at time t. This probability reads

$$\Pi_{i}(t)=(1-p)P_{i}(t)+p\sum_{j=1}^{N}R_{ij}P_{j}(t),$$ (5)

where the first term denotes the probability that a susceptible agent associated to patch i becomes infected when remaining at its home node i, and the second one accounts for the probability that this agent catches the disease when moving to any neighbor of i.

Finally, the probability $P_{i}(t)$ in Eq. (5) denotes the probability that a healthy agent in (but not necessarily associated to) node i at time t becomes infected after contact with any of the infected agents present inside i at the same time. Then, probability $P_{i}(t)$ reads

$$P_{i}(t)=1-\prod_{j=1}^{N}[1-\lambda \rho_{j}(t){]}^{n_{j\to i}},$$ (6)

where

$$n_{j\to i}=\delta_{ij}(1-p)n_{i}+pR_{ji}n_{j},$$ (7)

with $\delta_{ij}=1$ when i=j and $\delta_{ij}=0$ otherwise.

The expressions in Eqs. (4)–(7) compose the closed set of equations covering the evolution of a SIS disease spreading in the MIR metapopulation model with parameters p, μ, and λ. In addition, the matrix R is given by the topology of the mobility network, which can be constructed from the observed flows between the patches, and the set of node populations $\left\{n_{i}\right\}$ can also be set according to the local census of the population under study.

#### 2. SIR dynamics

The formulation of the Markovian equations for a metapopulation under a SIR spreading dynamics demands to add another set of N variables: $\left\{r_{i}(t)\right\}$ (i=1,…,N), i.e., the fraction of recovered agents associated to patch i. Thus, the set of N equations (4) for the SIS model is now substituted by the following set of 2N equations:

$$\rho_{i}(t+1)=(1-\mu )\rho_{i}(t)+[1-\rho_{i}(t)-r_{i}(t)]\Pi_{i}(t),$$ (8)

$$r_{i}(t+1)=r_{i}(t)+\mu \rho_{i}(t).$$ (9)

On the other hand, since the infection processes within each of the patches in the SIR model follow identical rules as those of the SIS one, the expression in Eq. (8) for the probability that a healthy agent associated to node i becomes infected at time t, $\Pi_{i}(t)$, has the same form as in the SIS case. This way, the SIR metapopulation dynamics is fully described by Eqs. (8) and (9) with the addition of Eqs. (5)–(7).

In Appendix A we illustrate the accuracy of the SIS and SIR Markovian equations by comparing their predictions with the results obtained via Monte Carlo numerical simulations in Erdös-Rényi (ER) and scale-free (SF) metapopulations.

## III. MULTIPLEX METAPOPULATIONS

After the former brief introduction to the Markovian formalism in monolayer metapopulations, we are ready to tackle the study of metapopulations in which different types of agents coexist and interact. The diversity of agents is manifested in their heterogeneous segregation across patches, so that the demographic partition into patches is independent for each class, and in their different mobility patterns. In particular, we focus on systems in which agents displaying L types of mobility patterns coexist within each patch. This way, the population of a patch i is the sum of the number of agents of each type $n_{i}=\sum_{\alpha =1}^{L}n_{i}^{\alpha }$ and the probability that an agent of patch i and type α visits another patch j is now written as the generalization of Eq. (1):

$$R_{ij}^{\alpha }=\frac{W_{ij}^{\alpha }}{\sum_{j=1}^{N}W_{ij}^{\alpha }},$$ (10)

where $W_{ij}^{\alpha }$ is associated to the number of observed trips of agents of type α in patch i to patch j.

**FIG. 2.**
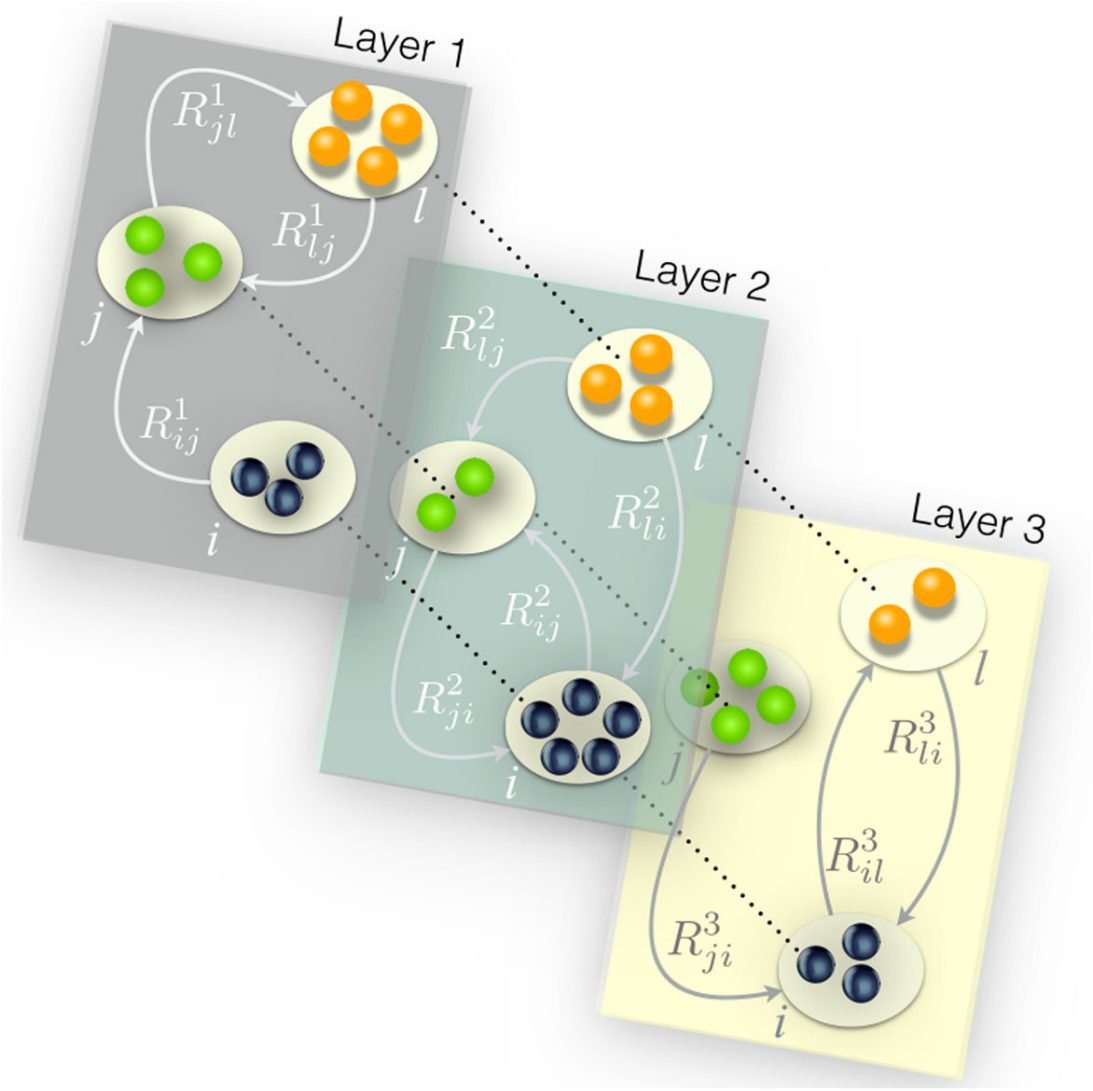
Schematic representation of a metapopulation multiplex composed of L=3 layers. Each of the N=3 patches (nodes) is represented in each of the layers. The layers highlight that individuals of type α associated to patch i move to another patch j with probability $R_{ij}^{\alpha }$ which, in general, is different from the rate of transitions of agents of type β≠α associated to the same node. This way, each network layer α presents a topology captured by a different matrix $R^{\alpha }$.

To analyze this situation, it is natural to make use of a multiplex formulation [31–35] of the metapopulation, as it is illustrated in Fig. 2. In our case, the number of layers of the multiplex is equal to the number of types of agents (L) and the architecture of each layer is described by a different matrix $R^{\alpha }$. Each patch of the system is represented as one node in each network layer, and the corresponding L nodes are virtually connected (dotted lines) as they mix their agents when the contagion processes take place.

The number of Markovian equations of the multiplex are now multiplied by L with respect to the networked metapopulation. In particular, for the SIS [and SIR] model, the variables are $\rho_{i}^{\alpha }(t)$ [and $r_{i}^{\alpha }(t)$], which denote the fraction of infected [and recovered] individuals of layer α=1,…,L associated to node i. In this case, SIR equations become

$$\rho_{i}^{\alpha }(t+1)=\rho_{i}^{\alpha }(t)(1-\mu )+\left[1-\rho_{i}^{\alpha }(t)-r_{i}^{\alpha }(t)\right]\;\,\left((1-p)P_{i}^{\alpha }(t)+p\sum_{j=1}^{N}R_{ij}^{\alpha }P_{j}^{\alpha }(t)\right),$$ (11)

$$r_{i}^{\alpha }(t+1)=r_{i}^{\alpha }(t)+(1-\mu )\rho_{i}^{\alpha }(t),$$ (12)

while for the SIS model we only have Eq. (11) with $r_{i}^{\alpha }(t)=0$. The term $P_{i}^{\alpha }(t)$, which denotes the probability that an agent of type α placed in patch i at time t becomes infected, reads

$$P_{i}^{\alpha }(t)=1-\overset{L}{\underset{\beta =1}{\prod }}\overset{N}{\underset{j=1}{\prod }}[1-\lambda^{\alpha \beta }\rho_{j}^{\beta }(t){]}^{n_{j\to i}^{\beta }(t)},$$ (13)

where $\lambda^{\alpha \beta }$ is the probability that a diseased agent of type β infects a healthy agent of type α. In addition, the number of agents of type α associated to patch j that travel to a different patch i is given by

$$n_{j\to i}^{\alpha }=(1-p)\delta_{ij}n_{i}^{\alpha }+pR_{ji}^{\alpha }n_{j}^{\alpha }.$$ (14)

The set of equations (11)–(14) conforms the Markovian model of the multiplex metapopulation. For the sake of simplicity, we now restrict to the case $\lambda^{\alpha \beta }=\lambda \;\;\forall \;\;\;\alpha$, β, so that the infection probability between healthy and infected agents does not depend on their types.

### A. Validation of the Markovian equations

To validate the Markovian equations for the multiplex metapopulation, we proceed in the same fashion as we did for networked ones. First, we compute the impact that SIR and SIS diseases have as a function of the infectivity of the disease λ and the degree of mobility p. We have studied three types of multiplex of L=2 layers, namely, ER-ER, SF-SF, and ER-SF, of $N=10^{3}$ nodes, and each node has an identical population of 500 agents. The weights of each link $W_{ij}^{\alpha }$ are randomly assigned following a homogeneous distribution in the range [1,39].

**FIG. 3.**
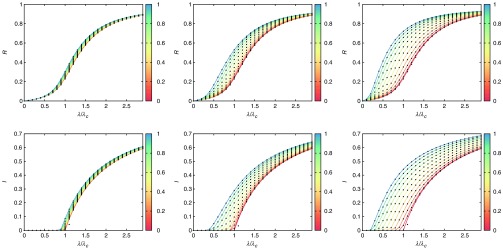
Epidemic diagrams for the SIR (top), R(λ) and SIS (bottom), I(λ) dynamics of three different multiplexes with L=2 layers. From left to right we have ER-ER, ER-SF, and SF-SF. In all the cases each network layer has $N=10^{3}$ nodes and each node contains 500 individuals per layer. The solid curves indicate the solution obtained by solving the Markovian evolution equations (the color of each curve indicates the value of p as shown in the color bars), whereas the points correspond to the results obtained by using agent-based simulations (50 realizations for each value of λ and p). Note that the value of λ has been rescaled by the critical value $\lambda_{c}$ at p=0, i.e., that of a well-mixed population of $n=10^{3}$ individuals: $\lambda_{c}=\mu /10^{3}$ at p=0. The recovery rate is μ=0.2.

In Fig. 3, we show the diagrams for the SIR (top) and the SIS (bottom) where dots represent the results obtained for Monte Carlo simulations of the epidemic processes and the solid lines are for the solution of the Markovian equations. As in the case of networked metapopulations, we observe a perfect agreement between simulations and the numerical solution of Eqs. (11)–(14). From the physical point of view we observe that, while for all the cases mobility enhances the anticipation of the epidemic onset, the multiplex composed of an ER and a SF topology yields an intermediate anticipation effect compared to those observed for ER-ER and SF-SF. This is an interesting result that differentiates what has been recently observed in epidemic processes in multiplex contact networks [40,41], where coupling L layers yields an overall epidemic threshold that is equal to the smallest threshold of the isolated layers or, in other words, the epidemic onset is driven by the largest of the maximum eigenvalues of the set of adjacency matrices that define the layers. It is clear that in the case of metapopulations the situation is more complicated as we show in the following section.

We now focus on the general scenario in which $\lambda^{\alpha \beta }\neq \lambda$, i.e., the contagion probability between two agents depends on their corresponding types. To this aim, we consider one population of agents whose movements are described by an ER mobility network and another population whose movements occur according to a SF graph. The number of patches is $N=10^{3}$, and inside each patch there are 500 agents of each type (ER and SF). We consider the situation in which $\lambda^{\alpha \beta }\ll \lambda^{\alpha \alpha }$ (α≠β). In particular, a contagion between agents moving in the ER layer occurs with probability $\lambda^{\mathrm{ER}}=1.5\mu /500$ and that for the agents moving in the SF layer is set to $\lambda^{\mathrm{SF}}=1.1\mu /500$ (recall that μ/500 is the epidemic threshold for a well-mixed population of 500 agents). In its turn, we have set the infection probability between agents of different type to $\lambda^{\mathrm{ER}\text{-}\mathrm{SF}}=\lambda^{\mathrm{SF}\text{-}\mathrm{ER}}=\;0.025\mu /500$. Finally, to work with a more heterogeneous setup, we study the case of a SIR dynamics in which a small seed of initial infected agents is set in a single patch and affects only agents of one type (here, those moving across the ER layer).

**FIG. 4.**
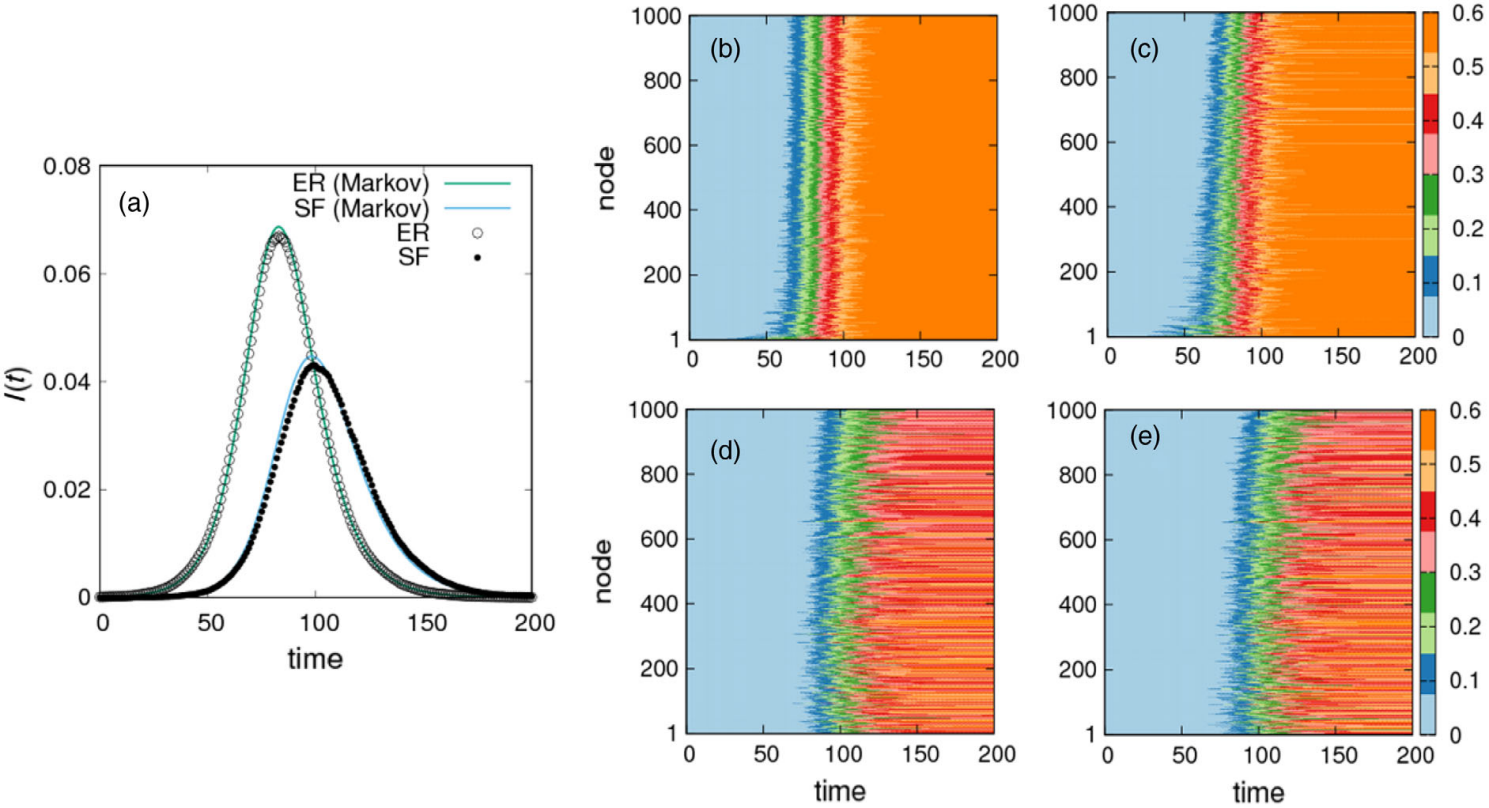
Spatiotemporal patterns of the SIR dynamics in a metapopulation multiplex composed of an ER and a SF layer. Each layer has $10^{3}$ patches and 500 individuals are associated to each patch. Theoretical prediction are shown by lines, whereas dots represent Monte Carlo results. The initial infected agents are placed in a single patch of the ER layer. This, together with the small contagion probability between agents of different layers (see the text for details), causes the time difference between the epidemic onsets in each layer as observed from (a). Panels (b)–(d) show the time evolution of the fraction of recovered agents for each patch. The top panels show this evolution in the ER layer obtained for Monte Carlo simulations (b) and the solution of the Markovian model (c). On the other hand, bottom panels show the same evolution in the SF layer as obtained again from simulations (d) and by solving the Markovian equations (11)–(14) (e).

To analyze the accuracy of Eqs. (11)–(14) in capturing the spatiotemporal evolution of epidemics, we first consider the temporal evolution of the fraction of infected individuals of each type (layer). In Fig. 4(a), we show this evolution comparing the solution of the Markovian equations (solid lines) with the result obtained from Monte Carlo simulations (points). It is clear that the Markovian equations (11)–(14) fairly reproduce the output of the numerical simulation, capturing the delay of the onset of the epidemics in the SF layer with respect to that in the population moving across the ER. This delay is a clear consequence of (i) the localization of the initial infected individuals in the ER layer and (ii) the small contagion probability between agents of different type (layer). Interestingly, the fact that $\lambda^{\mathrm{ER}\text{-}\mathrm{SF}}$ is far less than the threshold (μ/500) in a closed population of 500 agents does not prevent the disease from invading the SF layer.

Finally, in Figs. 4(b)–4(e), we show the temporal evolution of the fraction of recovered individuals for each patch in each of the layers (ER top and SF bottom) obtained from numerical simulations (left-hand panels) and solving Eqs. (11)–(14) (right-hand panels). The fair agreement between left- and right-hand panels indicates the great spatiotemporal accuracy of the Markovian model. Here, in addition to the delay in the onset of the epidemics in the SF population already observed in Fig. 4(a), it is remarkable that two different stationary regimes are obtained in each layer. Namely, the fraction of recovered individuals in the ER layer is nearly identical for all the patches. However, in the SF population the stationary pattern points out a far more heterogeneous distribution of recovered individuals across the different patches.

## IV. DEDUCTION OF THE EPIDEMIC THRESHOLD

The fair agreement between agent-based simulations and the solution of the Markovian equations allows us to make use of them in order to derive the analytical expression of the epidemic threshold. For the sake of simplicity, let us compute this value for the SIS case (similar results are obtained for the SIR model). In this case, see Eq. (11), the stationary solution for the fraction of infected agents of type α associated to patch i, $\rho_{i}^{\alpha \;⋆}$ fulfills

$$\mu \rho_{i}^{\alpha \;*}=(1-\rho_{i}^{\alpha \;*})\left((1-p)P_{i}^{\alpha \;*}+p\overset{N}{\underset{j=1}{\sum }}R_{ij}^{\alpha }P_{j}^{\alpha \;*}\right).$$ (15)

As usual for calculating the threshold, we linearize the above expression by considering that the fraction of infected people in the stationary state is very small ($\rho_{i}^{\alpha ⋆}=\varepsilon_{i}^{\alpha }\ll 1\forall \;\;\alpha \;\;\forall \;\;i$). This way, we can neglect second-order terms in $\varepsilon_{i}^{\alpha }$ in Eq (13), so that $P_{i}^{\alpha \;*}$ is given by

$$P_{i}^{\alpha \;*}=P_{i}^{*}=\overset{L}{\underset{\beta =1}{\sum }}\overset{N}{\underset{j=1}{\sum }}\lambda^{\alpha \beta }\varepsilon_{j}^{\beta }n_{j\to i}^{\beta }.$$ (16)

Introducing this expression into Eq. (15), the stationary state of the epidemics can be written as

$$\mu \varepsilon_{i}^{\alpha }=(1-p)\sum_{\beta =1}^{L}\sum_{j=1}^{N}\lambda^{\alpha \beta }\varepsilon_{j}^{\beta }(t)n_{j\to i}^{\beta }\;+p\sum_{j=1}^{N}R_{ij}^{\alpha }\sum_{\beta =1}^{L}\sum_{k=1}^{N}\lambda^{\alpha \beta }\varepsilon_{k}^{\beta }n_{k\to j}^{\beta }.$$ (17)

To incorporate the asymmetry between interlayer and intralayer interactions, we set the intralayer contagion probability to $\lambda^{\alpha \alpha }=\lambda$ while its interlayer counterpart reads $\lambda^{\alpha \beta }=\gamma \lambda$ (with γ∈[0,1] and α≠β). This way, the limit γ=0 describes the case of null interaction between agents of different types, whereas γ=1 recovers the indistinguishability of the agents type in terms of contagion processes. Under these premises, the general expression for the contagion probability $\lambda^{\alpha \beta }$ becomes

$$\lambda^{\alpha \beta }=\left[1-\left(1-\gamma \right)\left(1-\delta^{\alpha \beta }\right)\right]\lambda ,$$ (18)

where $\delta^{\alpha \beta }$ is the Kronecker delta, which is 1 if layers α=β, and 0 otherwise. Finally, by using the value of $n_{j\to i}^{\beta }$ from Eq. (14) and keeping up to first order in $\varepsilon_{i}^{\alpha }$, we obtain the expression

$$\frac{\mu }{\lambda }\varepsilon_{i}^{\alpha }=\overset{N}{\underset{j=1}{\sum }}\overset{L}{\underset{\beta =1}{\sum }}\underset{M_{ij}^{\alpha \beta }}{\underbrace{\left[1-\left(1-\gamma \right)\left(1-\delta^{\alpha \beta }\right)\right][(1-p{)}^{2}\delta_{ij}n_{i}^{\beta }+p(1-p)n_{j}^{\beta }(R_{ji}^{\beta }+R_{ij}^{\alpha })+p^{2}n_{j}^{\beta }(R^{\alpha }\cdot R^{\beta T}{)}_{ij}]}}\varepsilon_{j}^{\beta }.$$ (19)

At this point, it becomes clear that Eq. (19) defines an eigenvalue problem for the feasible solutions $\varepsilon_{i}^{\alpha }$. Indeed, there are N·L feasible solutions of λ corresponding to the eigenvalues of the N·L×N·L supramatrix M. However, since we are interested in the minimum value $\lambda_{c}$ for which Eq. (19) is fulfilled, the epidemic threshold is thus associated to the largest eigenvalue of M as

$$\lambda_{c}=\frac{\mu }{\Lambda_{\max}(M)}.$$ (20)

Let us now describe the entries of the matrix M, see Eq. (19), since they allow us to quantify the microscopical interactions among agents across the multiplex metapopulations. In fact, the elements $M_{ij}^{\alpha \beta }$ correspond, close to the epidemic threshold, to the probability that an agent of type α associated to patch i comes in contact with another one of type β from patch j. Specifically, each element contains three contributions accounting for the three potential sources of infections that a healthy agent can find: from agents associated to the same node inside this node [weighted by $(1-p{)}^{2}$], from agents from a different patch, either at one of the two patches they are associated to [weighted by p(1-p)], and from agents with whom they come in contact inside a third place different from their associated nodes (weighted by $p^{2}$).

To round out this derivation, let us remark that the generality of the expression for the epidemic threshold of multiplex metapopulations allows us to recover, by setting L=1, the value of the epidemic threshold in monolayer metapopulations. Indeed, for L=1, Eq. (19) turns into

$$\frac{\mu }{\lambda }\varepsilon_{i}^{*}=\overset{N}{\underset{j=1}{\sum }}\underset{M_{ij}}{\underbrace{\left[(1-p{)}^{2}\delta_{ij}n_{j}+p(1-p)n_{j}{\left(R+R^{T}\right)}_{ij}+p^{2}n_{j}{\left(R\cdot R^{T}\right)}_{ij}\right]}}\varepsilon_{j}^{*},$$ (21)

so that the epidemic threshold is given by

$$\lambda_{c}=\frac{\mu }{\Lambda_{\max}(M)},$$ (22)

where M is now an N×N matrix. In the same fashion as supramatrix M, each term $M_{ij}$ of matrix M encodes the probability that an agent associated to patch i comes in contact with another from patch j.

**FIG. 5.**
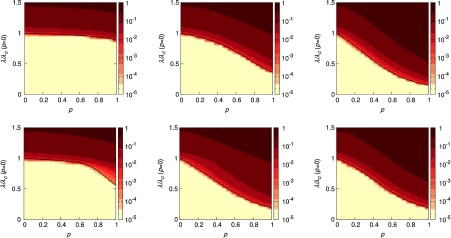
Epidemic diagrams I(λ,p) for SIS dynamics over those multiplex metapopulations used in Fig. 3. The color code (see color bar) denotes the fraction of infected individuals in the steady state as obtained from Monte Carlo simulations. The solid curves indicate the function $\lambda_{c}(p)$ as dictated from Eq. (20) for the multiplex metapopulations. The values of interlayer coupling are γ=1 (top) and γ=0 (bottom). The recovery rate of the SIS dynamics has been set to μ=0.2. From left to right we have used ER-ER, ER-SF, and SF-SF architectures to simulate the evolution of a disease.

We have checked the validity of Eq. (20) by computing the largest eigenvalue of M for the three synthetic multiplexes under study in Fig. 3 for a range of values of p∈[0,1]. This way, through Eq. (20) we obtain a curve $\lambda_{c}(p)$, see Fig. 5, that reproduces the onset observed in Monte Carlo simulations for both indistinguishable agents γ=1 (top) and noninteracting layers γ=0 (bottom). The monotonous decrease of $\lambda_{c}(p)$ corroborates that, for these three synthetic multiplexes, mobility enhances the spread of the disease. Interestingly, for the case of noninteracting layers, the epidemic threshold of the ER-SF and SF-SF multiplexes follows the same dependence on the mobility. This result indicates that it is the layer with the smallest threshold, the SF one (see Fig. 12 in Appendix A), the one driving epidemic outbreaks. However, as the interlayer coupling increases, the two layers interplay and the ER-SF and SF-SF metapopulations behave differently. Interestingly, for γ=1 the effect of the ER layer in the ER-SF multiplex is to soften the trend of the epidemic threshold with the mobility compared to γ=0.

## V. REAL MULTIPLEX METAPOPULATIONS DETERMINED BY SOCIOECONOMIC CLASSES

The formalism proposed here offers the possibility of accounting for the coexistence of different mobility patterns within the inhabitants of real populations. This possibility allows us to gain insights about the role played by the interactions between different kinds of agents in spreading processes. To shed light on the applicability of this formalism in real populations and to fully exploit the possibilities offered by the multiplex formulation, we now study the SIS and SIR spreading dynamics in a real urban system, the city of Medellín (Colombia), where six different socioeconomic classes coexist. Specifically, these social classes range from 1, which includes those inhabitants with the lowest incomes, to class 6, corresponding to the wealthiest individuals. The separation into six socioeconomic classes in Colombia [42] and, in particular, in large cities such as Medellín (the second largest city in Colombia with around $5\times 10^{6}$ inhabitants) leads to a different demographic distribution across towns and, equally important, to different mobility patterns due to their heterogeneous needs and transportation services at hand (see Refs. [43,44] for details).

**FIG. 6.**
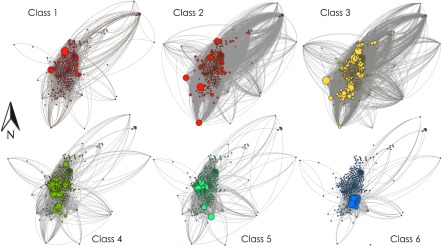
Mobility networks of each socioeconomic class in the city of Medellín. Each panel shows the geographical location of each subpopulation (node) in the city of Medellín. The size of each node is proportional to the number of agents of the corresponding socioeconomic class in the subpopulation. The connections between two nodes denotes the existence of back-and-forth movements between two subpopulations for a given socioeconomic class.

To study the evolution of diseases while preserving the information related to the existence of different socioeconomic classes, we make use of the former formalism by constructing, from the data presented in Refs. [45,46], a multiplex network of six layers. As shown in Fig. 6, each layer contains the specific recurrent mobility patterns of each socioeconomic class among the 413 areas (nodes) in which the city of Medellin is divided. Note that, apart from the different link patterns of the layers, the distribution of the agents across the 413 areas depends strongly on the particular socioeconomic class. For instance, it is clear that agents belonging to the lowest income class 1 tend to localize in northern areas of the city, whereas individuals of class 6 concentrate in those areas in the south.

### A. Epidemic incidence on social classes

**FIG. 7.**
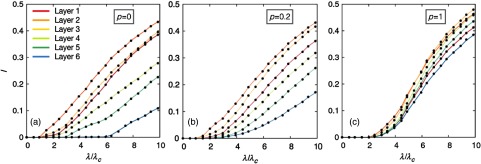
Impact of a SIS disease I(λ) on each of the layers of a real multiplex metapopulation. Note that the value of λ has been rescaled by the critical value $\lambda_{c}$ at p=0. The mobility of the agents has been set from left to the right to p=0, p=0.2, and p=1. Solid lines correspond to theoretical predictions obtained by iterating Eqs. (11)–(14), whereas black dots are the result from averaging 20 realizations of numerical simulations. The recovery rate of both dynamics has been set to μ=0.2.

In this section, we aim at quantifying the impact for each socioeconomic class of a disease propagated across the city of Medellín. For the sake of simplicity, let us first consider the case of indistinguishable interacting agents, i.e., γ=1. As for the case of synthetic networks, we show in Appendix B the accuracy of our formalism in capturing the global incidence of SIS and SIR diseases. The epidemic incidence for each socioeconomic class is shown in Fig. 7 by plotting, for several values of the mobility p, the epidemic diagrams for a SIS disease, I(λ). Apart from the fair agreement between the Markovian formulation and Monte Carlo simulations, we observe that, regardless of the value of p, layer 2 drives the onset of epidemics in the multiplex whereas layer 6 (the wealthiest class) turns out to be the least affected by the disease.

Some features about the underlying multiplex network can be inferred from these graphs. For instance, the results corresponding to the static case (p=0) unveil the demographic distribution of the layers. On the one hand, in Fig. 7(a), we observe that agents from classes 1, 2, and 3 occupy the most populated nodes, since the epidemic onset associated to these layers is the smallest one. On the other hand, it becomes clear that individuals from class 6 reside practically isolated from the rest of the classes, occupying sparsely populated neighborhoods. Additionally, from Figs. 7(b) and 7(c), we notice the balancing role of mobility: by increasing p, social mixing is boosted and, as a consequence, the epidemic incidence in the layers become more similar.

**FIG. 8.**
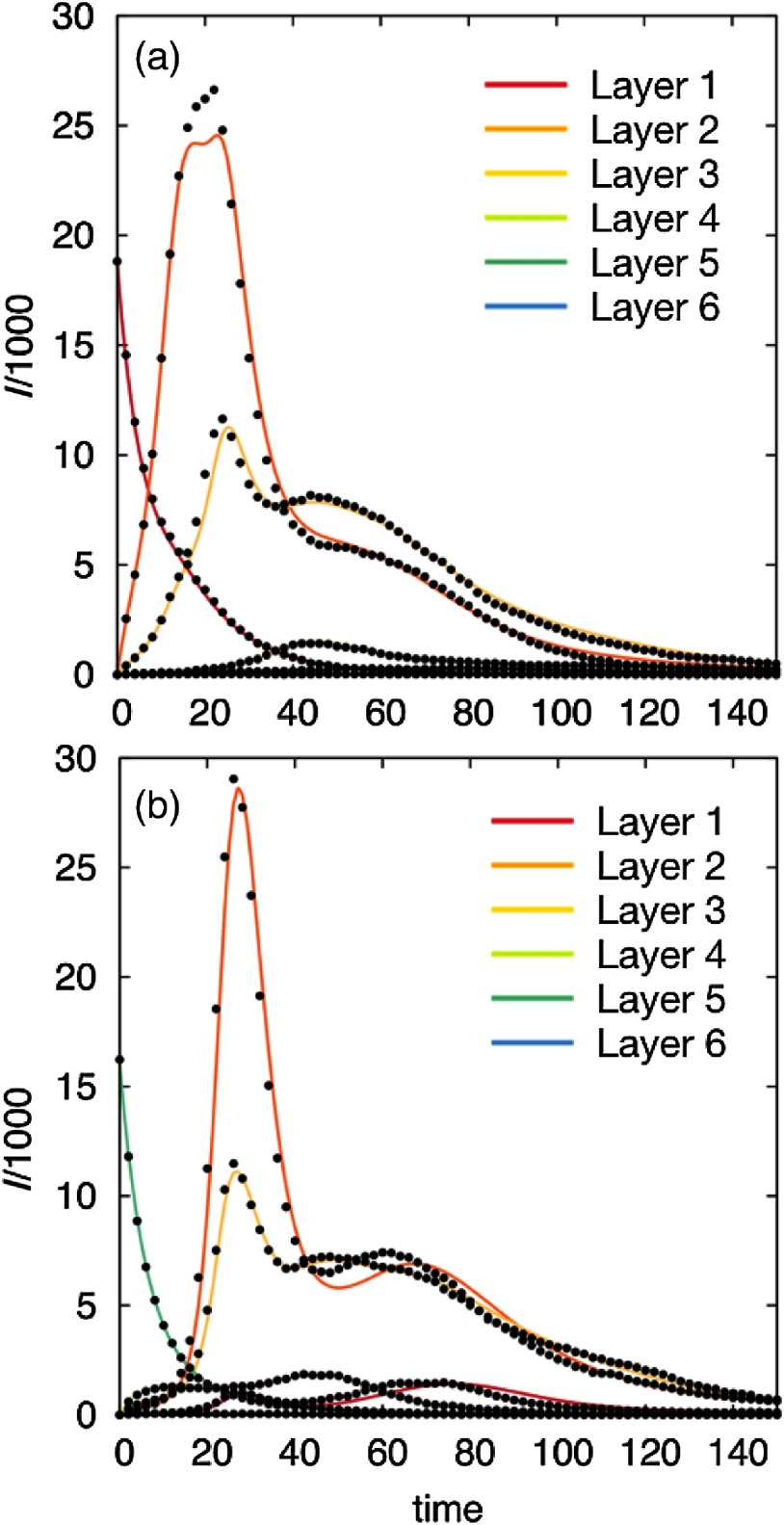
Temporal evolution of a SIR disease whose seed is initially localized inside layer 1 (top) and layer 5 (bottom). Solid lines correspond to theoretical predictions according to Eqs. (11)–(14), whereas black dots are the output of Monte Carlo simulations. The mobility of the agents p, the contagion rate λ, and the recovery rate μ have been set to ($p,\lambda ,\mu )=[0.05,4\lambda_{c}(p=0),0.2]$.

To get more insight about the interaction among the different layers and to further validate our formalism, we now address the spatiotemporal propagation of diseases whose initial seed is localized inside one of the layers. For this purpose, we have fixed the parameters of our model (p, λ, μ) and represented in Fig. 8 the time evolution of the number of infected agents according to a SIR disease for each socioeconomic class when the seed is localized in classes 1 [Fig. 8(a)] and 5 [Figs. 8(b)]. Again, we also compare the results of the Markovian evolution equations (curves) with Monte Carlo agent-based simulations (points).

The solution of the Markovian equations captures the nontrivial interaction patterns between the different socioeconomic classes. In particular, it can be noticed that contagion processes take place mainly among close classes (in terms of incomes) since they show a cascadelike structure: 1→2, 3→4 in Fig. 8(a) and 5→4→3→4, 2→1 in Fig. 8(b). Finally, the nontrivial nature of the time evolution of infections is captured by the existence of a feedback phenomenon when looking to the sequence of local outbreaks for classes 2, 3, and 4. The observed correlations between layers’ outbreaks reveals the closeness between the individuals in these middle-class layers.

### B. Epidemics and interlayer coupling

Up to this point, we have assumed that contagion processes between agents from Medellín do not depend on the layers (socioeconomical class) to which interacting agents belong. However, in real systems, the social mixing between different socioeconomical classes is far from homogeneous, being more typically contacts between agents of the same or similar socioeconomical classes. Thus, in multiplex terminology, the assumption that an agent interacts in the same way with agents of the same layer and with those of different layers, $\lambda^{\alpha \beta }=\lambda$, is no longer a valid premise. To analyze the role of social mixing between the different socioeconomic classes, we make use of the interlayer coupling γ and analyze the behavior of the epidemic threshold $\lambda_{c}$ as γ varies from 0 (noninteracting classes) to 1 (fully indistinguishable classes).

**FIG. 9.**
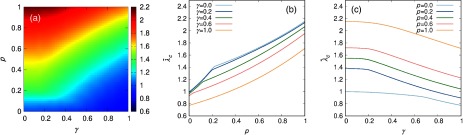
(a) Epidemic threshold (color code) as a function of the agents’ mobility p and the interlayer coupling γ. (b) Epidemic threshold as a function of the mobility for several values of the interlayer coupling. (c) Epidemic threshold as a function of the interlayer coupling for several values of the mobility. Note that the epidemic threshold has been rescaled by the epidemic threshold corresponding to the static case and decoupled layers, so that ${\tilde{\lambda }}_{c}=\lambda_{c}(p,\gamma )/\lambda_{c}(0,0)$.

To illustrate the applicability of Eq. (20) in a real case, let us focus on the analysis of the role that socioeconomic mixing has on the epidemic threshold for the city of Medellín. In Fig. 9(a), we show the surface $\lambda_{c}(p,\gamma )$ calculated from the supramatrix $M^{\prime }$ obtained from the data of the city of Medellín. From this surface it becomes evident that an increase of social mixing γ always leads to a decrease of the epidemic threshold since γ promotes the number of contacts taking place inside each subpopulation. Regarding the role that mobility plays, it becomes clear that, for all the values of the interlayer coupling, increasing the agents’ movements always has a detrimental effect on the onset of epidemics, which is identified by an increase of the epidemic threshold. Both factors then can counterbalance each other and will be responsible of interesting nontrivial effects on the criticality of the epidemics. The pattern observed in the surface $\lambda_{c}(p,\gamma )$ points out a nontrivial dependence of the epidemic threshold with the mobility for small values of the social mixing γ. This dependence is better visualized in Figs. 9(b) and 9(c), where we show the curves $\lambda_{c}(p)$ for several γ values and their counterpart, i.e, the curves $\lambda_{c}(\gamma )$ for several values of p.

Interestingly, for low values of γ, at certain values of the mobility $p^{\prime }$ a sharp change in the slope of $\lambda_{c}(p)$ takes place. Given the dependence of $\lambda_{c}$ on $\Lambda_{\max}\left(M^{\prime }\right)$ this sudden variation is associated to a change on the leading eigenvector of $M^{\prime }$. This phenomenon is in close analogy to the findings by Ref. [35] for the spectra of the supra-Laplacian matrix, where the change on the order of the relevant eigenvalues leads to an abrupt change of the critical properties of the multiplex. In the following section, we present a deeper analysis of this phenomenon and its implications.

### C. Outbreak localization transitions

To get insight on the abrupt change of tendency in the evolution of the epidemic threshold observed in Figs. 9(b) and 9(c) for certain values of (p, γ) = $\left(p^{\prime },\gamma^{\prime }\right)$, we analyze the structure of the leading eigenvector of matrix $M^{\prime }$. The components of the leading eigenvector $v_{\max}$ corresponding to $\Lambda_{\max}\left(M^{\prime }\right)$ encode those subpopulations driving the onset of the epidemic close to the epidemic threshold. If the structure of this eigenvector $v_{\max}$, which controls the onset of epidemics, also changes at $\left(p^{\prime },\gamma^{\prime }\right)$, it implies that the contribution of each subpopulation to the epidemic onset is eventually altered.

The analysis of the components of $v_{\max}$ reveals that this is indeed the case. The distribution of values of the components of $v_{\max}$ shows different localizations, i.e., significantly larger contributions of different subpopulations, as a function of the mobility parameter p and the social mixing controlled by γ. The existence of different localizations $v_{\max}$ depending on the mobility is crucial for designing efficient policies to ameliorate the onset of diseases since the particular contribution of each subpopulation encoded in the components of $v_{\max}$ allows us to apply targeted immunization strategies. Specifically, as the patches in the metapopulation of Medellín correspond to neighborhoods, identifying the largest components of $v_{\max}$ helps us to identify the most critical urban areas. To do so, note that first we must filter those entries of the matrix ${M^{\prime }}_{ij}^{\alpha \beta }$ which are physically infeasible, i.e., having zero individuals in a patch i in layer α. This filtering is essential to make predictions about real epidemic scenarios and does not have any influence on its eigenvalues, so that there are no changes in the predictions about the epidemic threshold. Once we have filtered out these artifacts, we compute the leading eigenvector of the matrix $M^{\prime }$.

A way to quantify the former description is focusing on the overall contribution of each geographical patch to $v_{\max}$ across all layers. This can be achieved simply by coarse graining the eigenvector summing the contributions of each layer associated to the same urban area i, obtaining a new eigenvector $V_{\max}$ of N entries which are given by

$${\left(V_{\max}\right)}_{i}=\frac{\overset{L}{\underset{\alpha =1}{\sum }}{\left(v_{\max}\right)}_{i}^{\alpha }}{\sqrt{\overset{N}{\underset{j=1}{\sum }}[\overset{L}{\underset{\alpha =1}{\sum }}{\left(v_{\max}\right)}_{j}^{\alpha }{]}^{2}}},$$ (23)

where the denominator accounts for the normalization of the projected eigenvector $V_{\max}$.

**FIG. 10.**
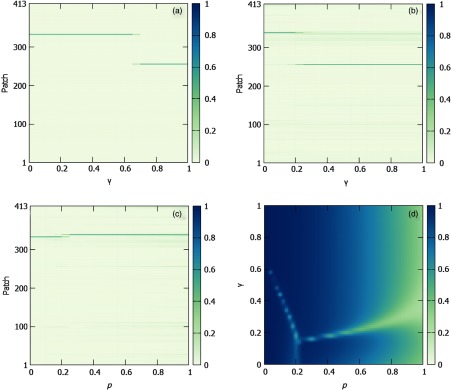
(a),(b) Magnitude of the components of the leading eigenvector (color coded) as a function of the social mixing γ for every subpopulation (patch); see Eq. (23). The mobility of the agents has been set to (a) p=0.0 and (b) p=0.6. (c) Same as (a) and (b), but now fixing the interlayer coupling to γ=0.1 and monitoring the evolution of the leading eigenvector with p. (d) Inverse participation ratio (IPR) (color code) according to Eq. (24) as a function of p and γ. Note that there are abrupt changes in the IPR for certain values of (p, γ). These strong variations encode the delocalization processes that take place when the dominant patch, which triggers the epidemic onsets, changes.

In Figs. 10(a) and 10(b), we show the evolution of the projected eigenvector with γ assuming that agents’ mobility is p=0 (a) and p=0.6. In these cases, the eigenvectors are pretty localized in a few patches, pointing out that targeted policies should be implemented to control epidemic outbreaks. Moreover, as anticipated before, strong variations in the leading eigenvector components occur while varying social mixing γ. For the chosen mobility values, these transitions take place for $\gamma^{\prime }≃0.63$ when p=0 and $\gamma^{\prime }≃0.21$ when p=0.6. For the sake of completeness, we have also represented in Fig. 10(c) a complementary figure by fixing γ=0.1 and modifying agents’ mobility p. In this case, we also find a sharp transition in the leading eigenvector which occurs for $p^{\prime }≃0.18$. Note that, as a direct consequence of the current findings, containment strategies targeting a certain patch can pass from efficient to useless under small changes in either the agents’ mobility or their social mixing.

Finally, to have a more general and explicative picture of the phenomena described above, we compute the inverse participation ratio (IPR) of the projected eigenvector $V_{\max}$ as a function of the agents’ mobility p and the interlayer coupling γ. This quantity has been proved to be very useful for studying the localization of spreading dynamics in complex networks [47,48]. In our case, this quantity reads as follows:

$$\mathrm{IPR}=\sum_{i=1}^{N}{\left(V_{\max}\right)}_{i}^{4},$$ (24)

where ${\left(V_{\max}\right)}_{i}$ is given by Eq. (23). This definition bounds IPR between IPR=1/N, corresponding to a completely delocalized state, and IPR=1, for which the eigenvalue is strictly localized in one patch.

In Fig. 10(d), we show the inverse participation ratio as a function of the mobility and the social mixing. It can be observed that this indicator captures the transition points previously reported as sudden changes in the localization of the leading eigenvector of the matrix $M^{\prime }$ for small values of p and γ. These sudden changes consist of abrupt decreases of the IPR, pointing out the delocalization processes necessary to move from one localized eigenvector to another one localized in the other node. Finally, as p increases, for all γ values, a drop in IPR occurs due to the delocalization of the eigenvector components because of the geographical mixing provided by the large mobility of agents.

### D. Physical interpretation of the abrupt changes in the populations triggering epidemics

We now try to understand the physical roots beneath the abrupt changes in the components of the leading eigenvector of matrix M. To shed light on these phenomenon we must identify those critical patches driving the epidemic onset without performing any spectral analysis. The most logical way to tackle this problem is to compute the total number of contacts performed by the agents of a given patch i and socioeconomic class α. This way, we can identify the urban area and class whose inhabitants are more likely to contract the disease due to their higher participation in contagion processes. At this point, let us recall the physical meaning of the entries of matrix M, Eq. (19). In particular, $M_{ij}^{\alpha \beta }$ encodes all the possible contacts between one individual from patch i at layer α and agents from patch j at layer β. This way, the effective number of contacts of agents from i at α can be computed as

$$C_{i}^{\alpha }(p,\gamma )=\sum_{\beta =1}^{L}\sum_{j=1}^{N}M_{ij}^{\alpha \beta }(p,\gamma ).$$ (25)

**FIG. 11.**
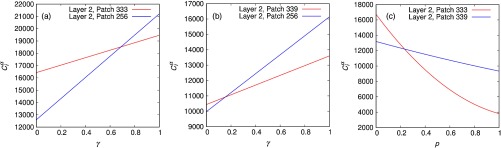
(a),(b) Average number of contacts $C_{i}^{\alpha }$ in which agents from patch i at layer α participate as a function of γ. For the sake of clarity, only the largest values of this indicator, which are relevant for identifying the critical patches, have been represented. The chosen values of the mobility are p=0 in (a) and p=0.6 in (b). (c) Average number of contacts $C_{i}^{\alpha }$ in which agents from patch i at layer α participate as a function of the mobility for γ=0.1.

**FIG. 12.**
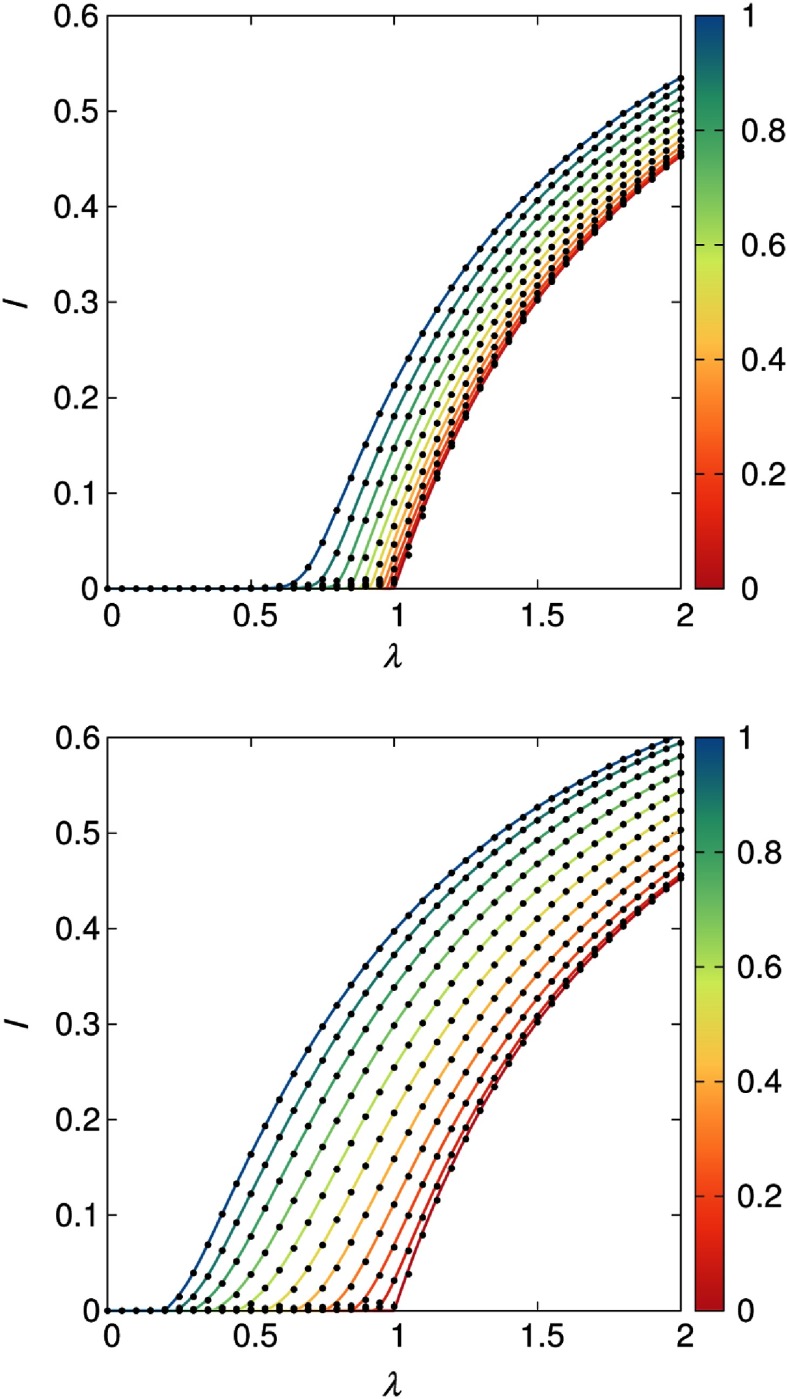
I(λ) for the SIS dynamics in ER (top) and SF (bottom) networks of $10^{3}$ nodes and ⟨k⟩=5.5 and ⟨k⟩=7.3, respectively. The population of each node is $5\times 10^{3}$ individuals. The solid curves indicate the solution obtained by solving the Markovian evolution equations (the color of each curve indicates the value of p as shown in the color bars), whereas the points correspond to the results obtained by using Monte Carlo simulations (20 realizations for each value of λ). Note that the value of λ has been rescaled by the critical value at p=0, i.e., that of a well-mixed population of n=5000 individuals: $\lambda_{c}(p=0)=\mu /n=4\times 10^{-4}$. The recovery rate is μ=0.2.

To illustrate the applicability of this quantity to identify those areas that are more likely to trigger the epidemic outbreak, let us represent, see Fig. 11, the two largest values of the effective number of contacts as a function of (p, γ) for the cases depicted in Fig. 10. Interestingly, these two largest values of $C_{i}^{\alpha }$ correspond to the patches involved in each of the abrupt transitions reported in Fig. 10, thus revealing that the effective number of contacts captures those driver nodes that appear in the leading eigenvector of M. Moreover, the effective number of contacts also captures those values $\gamma^{\prime }$ and $p^{\prime }$ where the abrupt transitions in the leading eigenvector take place. As a consequence, we can physically explain the observed transitions by computing the values of the mobility and the interlayer coupling for which the contacts of agents from one patch and layer surpass those corresponding to the former dominant patch.

## VI. CONCLUSIONS

In this work, we elaborate a theoretical formalism to analyze spreading processes in multiplex metapopulations characterized by recurrent mobility patterns. Our framework gets rid of the assumptions about the correlations between the node attributes and epidemic variables introduced in heterogeneous mean-field formulations. This way, the formalism introduced here is general enough so as to accommodate any origin-destination (weighted and directed) matrix containing different commuting patterns within a population and to cast the information about the local census of each patch.

First, we introduce the Markovian evolution equations for the monoplex (single-layer multiplex) case under the SIR and SIS dynamics. The second step is to generalize the former formalism to address metapopulations composed of several types of agents whose mobility patterns are different. To this aim, we make use of the multiplex formalism, thus constructing a multiplex metapopulation. We check the validity of the Markovian formalism by solving the equations and comparing their solution with the results obtained from Monte Carlo simulations in synthetic multiplex metapopulations. The agreement we obtain is remarkable both at the macroscopic and the microscopic level, even reproducing the spatiotemporal epidemic patterns capturing the onset of epidemics at the local level of patches.

The validity of the Markovian equations has allowed us to derive analytical expressions for the global epidemic threshold of multiplex metapopulations. Again, the analytical prediction is in complete agreement with numerical simulations. Interestingly, the onset is related to the maximum eigenvalue of a supramatrix M in which the different mobility patterns, local census, the degree of mobility, and the social mixing interplay. Remarkably, the structure of the supramatrix M captures three basic contagion processes for a healthy individual.

On more general grounds, dynamical processes on multiplexes have been a research focus in recent years [39,49–52], along with, in particular, their application to epidemics [53]. As usual in the multiplex literature, the scenario considered is that of coupled contact networks, so that a node is an individual that interacts in different ways (i.e., through different interaction layers) with the rest of the nodes. Under this setting, different problems, such as the diffusion of a disease through different contagion channels [40,41,54], the cooperative spreading of different diseases [55–58], or the coevolution of different contagion processes [59–61], have been addressed. Here, at variance, the two interaction levels (epidemics and mobility) of the metapopulation yield interesting results related to the interplay of the architecture of layers.

Finally, we show the applicability of the formalism to a real case study: the city of Medellín (Colombia). To this end, we gathered data of the mobility patterns for different socioeconomical classes (the layers of the multiplex). The first interesting result is the presence of epidemic detriment with mobility [26] for the full multiplex structure while, for each individual layer, the phase diagram does not show this phenomenon. Additionally, the multiplex formalism allows us to study the mixing among the different socioeconomic classes in Medellín using a single parameter (intensity of the interlayer link). An exhaustive analysis of the epidemic threshold reveals that, when mixing between different socioeconomic classes is small, there is a sudden change in the localization components of the eigenvector controlling the epidemic onset as mobility increases. This transition implies that the set of subpopulations triggering the spread of the disease changes abruptly, and can be detected. Moreover, we derive an indicator that is the effective number of contacts of the agents of one agent from a given patch and class. This indicator, which only depends on the underlying multiplex metapopulation, allows us to determine the drivers nodes and, more importantly, the transition points where the abrupt changes in the localization of epidemic outbreaks occur. These results point out that the multiplex nature of urban systems and the interplay between mobility and the social mixing of their inhabitants must be carefully taken into account in order to design efficient containment policies.

In a nutshell, the formalism we introduce here provides us with a reliable and computationally time-saving platform to analyze the epidemic risk of systems displaying recurrent mobility patterns. This way, the formalism can be used to readily identify those critical areas that spur the unfolding of diseases. In addition, the possibility of handling analytical equations can be further exploited beyond the derivation of the epidemic threshold and combined together with control techniques to test in an efficient way different contention policies. We expect as well that our Markovian formalism can be further extended in the future to accommodate more sophisticated commuting patterns and more refined epidemic models, thus better representing real epidemic scenarios.

## Figures and Tables

**FIG. 13.**
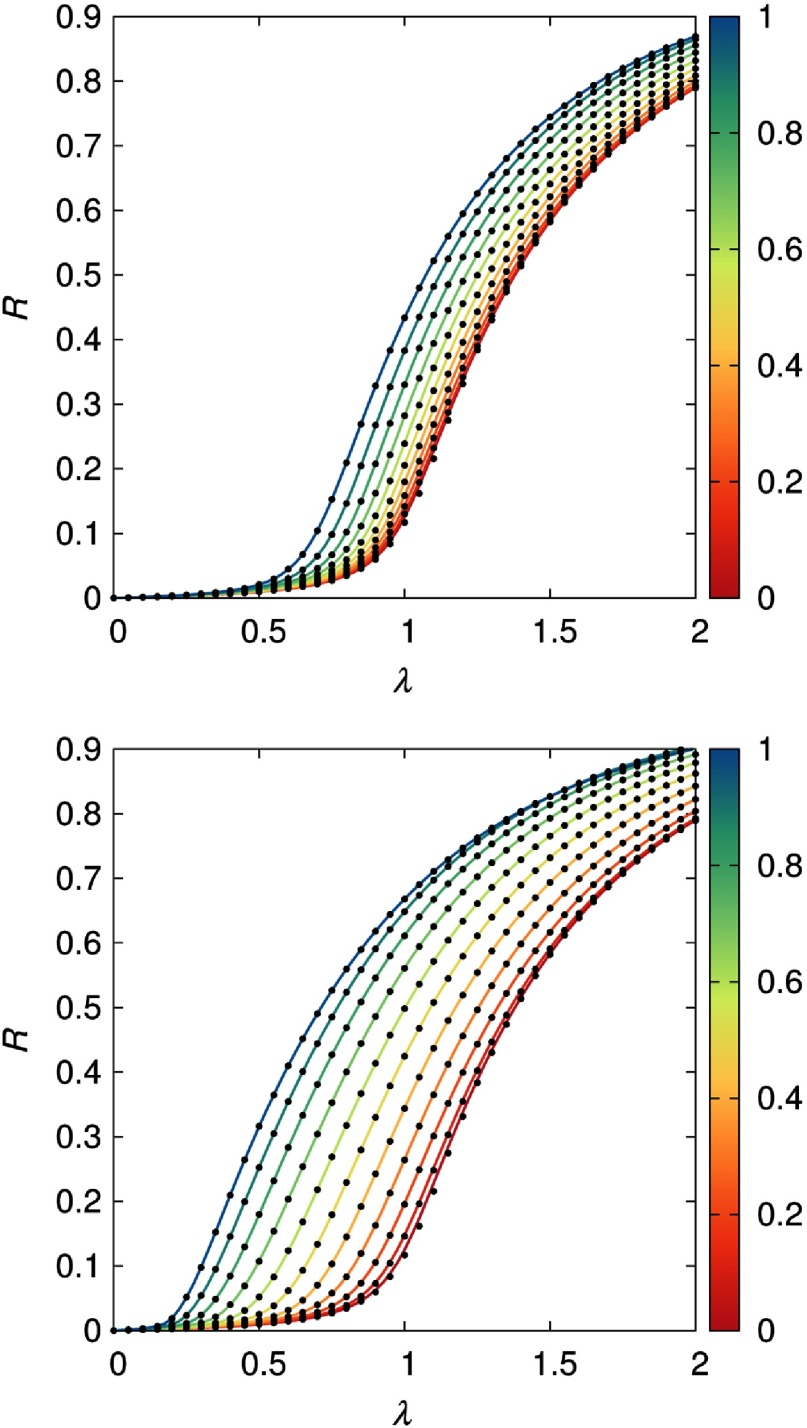
R(λ) for the SIR dynamics in ER (top) and SF (bottom) networks of $10^{3}$ nodes and ⟨k⟩=5.5 and ⟨k⟩=7.3, respectively. The population of each node is 5000 individuals. The solid curves indicate the solution obtained by solving the Markovian evolution equations (the color of each curve indicates the value of p as shown in the color bars), whereas the points correspond to the results obtained by using Monte Carlo simulations ($10^{2}$ realizations for each value of λ). Note that the value of λ has been rescaled by the critical value at p=0, i.e., that of a well-mixed population of $n=5\times 10^{3}$ individuals: $\lambda_{c}(p=0)=\mu /n=4\times 10^{-4}$. The recovery rate is μ=0.2.

**FIG. 14.**
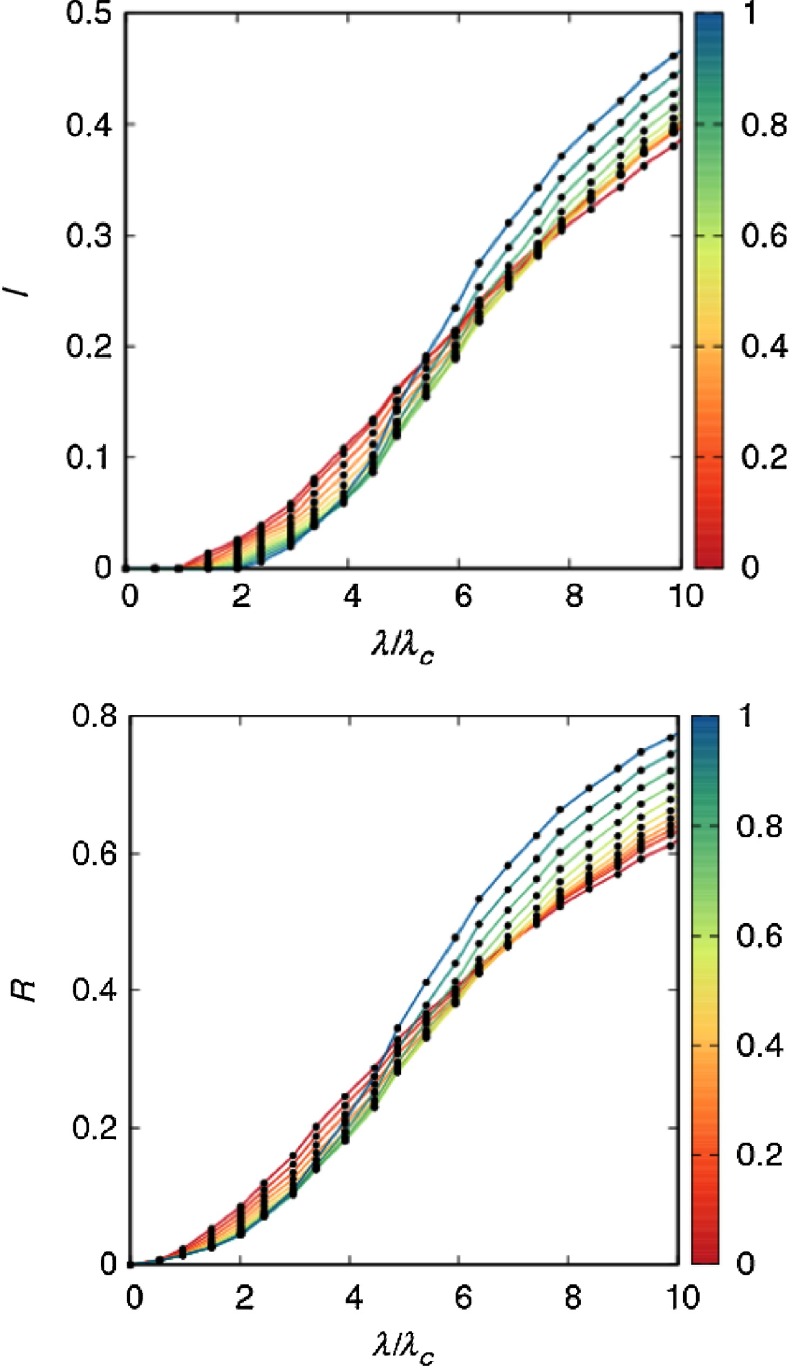
Epidemic diagrams I(λ) (top) and R(λ) (bottom) for SIR and SIS dynamics, respectively, in a real multiplex metapopulation. Solid lines denote the predictions of our model about the incidence of a disease, whereas black dots show the results obtained from averaging 20 realizations of numerical simulations. The color code denotes the value of the mobility of the agents p. The recovery rate is set to μ=0.2.

## APPENDIX A: VALIDATION OF THE MARKOVIAN EQUATIONS FOR SYNTHETIC SINGLE METAPOPULATIONS

To check the accuracy of the Markovian equations we have considered ER and SF synthetic networks having the same number of nodes $N=10^{3}$ and average connectivities ⟨k⟩=5.5 and ⟨k⟩=7.3, respectively. The nodes of these networks are homogeneously populated, $n_{i}=5\times 10^{3}\;\;\forall \;\;i$, so that the total population of our systems is $P=5\times 10^{6}$. The weights $W_{ij}$ between the nodes of the graphs are randomly assigned following a homogeneous distribution within the range $W_{ij}\in [1,50]$. Once all the weights are set, we construct the transition matrix R [see Eq. (1)] for each graph.

Monte Carlo simulations start by infecting a small fraction of agents in each of the nodes. In particular, we infect each agent with probability $10^{-3}$ so that, on average, there is 1 infected agent per node at time t=0. This initial configuration corresponds to set as initial conditions of the Markovian equations $\rho_{i}(0)=10^{-3}\;\;\forall \;\;i$ [and $r_{i}(0)=0\;\;\forall \;\;i$ in the SIR case]. For Monte Carlo simulations, due to the stochastic nature of the initial configuration and the disease models, we have averaged the results over $10^{2}$ realizations for each combination of the parameters (p, λ, and μ) considered.

First, we analyze the SIS model. In Fig. 12 (top and bottom panels correspond to ER and SF networks, respectively), we plot the number of infected agents in the steady state I as a function of the infection probability λ for different movement probabilities p. The points denote the results of Monte Carlo simulations for each value of λ and p while solid curves correspond to the solution of the Markovian equations. The agreement between simulations and the equations is almost exact, capturing with high accuracy the macroscopic state of the metapopulation in both the disease-free and epidemic regimes. For the SIR model in Fig. 13 (top and bottom panels correspond to ER and SF networks, respectively), we plot the number of recovered agents R as function of λ for the same set of movement probabilities p as for the SIS model. Again, we observe the exact agreement between Monte Carlo simulations and the solution of Markovian equations both before and after the epidemic threshold.

The high accuracy of the solution of Markovian equations shown in Figs. 12 and 13 allows us to overcome the computational costs associated to large-scale Monte Carlo simulations. However, it is clear that both I (SIS) and R (SIR) are macroscopic indicators of the outreach of the disease in the whole population. The examples shown above assume that the populations across nodes are homogeneously distributed. However, in real metapopulations, such as cities, each patch contains a different number of agents. These demographic heterogeneity may lead to interesting effects, such as the increase of the epidemic threshold with the increase of mobility [26,27]. Here, due to the homogeneous distribution of agent across patches, mobility always leads to a decrease of the epidemic onset (as shown in Figs. 12 and 13).

## APPENDIX B: VALIDATION OF THE MARKOVIAN EQUATIONS FOR MEDELLÍN MULTIPLEX

Finally, we check the accuracy of the Markovian equations in predicting the impact of epidemics on the city of Medellín at a global scale. In Fig. 14, we plot the epidemic diagrams corresponding to different values of the degree of mobility p for both SIS and SIR diseases. These diagrams are obtained via Monte Carlo simulations (points) and by solving the Markovian equations (lines), showing an excellent agreement. Interestingly, we can also notice that, in Medellín, mobility hinders epidemic onsets, since the epidemic threshold increases with p. This counterintuitive behavior, already reported for monolayer configurations [26], emerges from the homogenization of the demographic distribution across urban areas as mobility increases.
